# Abstract of the Procedings of the Buffalo Medical Association, Dec. 7th, 1875

**Published:** 1876-01

**Authors:** E. N. Brush

**Affiliations:** Secretary


ART. III.—*Abstract of the Proceedings of the Buffalo Medical Association, Tuesday Evening, Dec. 7th,* 1875. Reported by E. N. BRUSH, M. D., Secretary.

The President, Dr. GOULD, in the chair.

Members present: Drs. Cronyn, Harvey, Howe, O'Brien, Samo, Boysen, Hopkins, Fowler, Lothrop, Abbott, Brecht, Little, Wyckoff, Barnes, Folwell and McNiel.

By invitation Dr. Shannon, U. S. A., Dr. W. Cronyn, U. S. N., and Dr. Chester.

Dr. HOPKINS red a paper on "Exercise as a Remedial Agent."*

Dr. HOWE exhibited an apparatus for protecting the eye at night from injury by the hands of the patient after operations, etc. The apparatus consisted of an elipsoidal wire mask some three inches in its long diameter, the edges bent to adapt it to the form of the face. It is fastened about the head by means of an elastic band, effectually preventing the patient from touching his eyes in any manner during sleep.

Dr. CRONYN said he did not think that Dr. Hopkins' excellent paper should be passed without notice. Theoretically the subject is of value, and Dr. Hopkins has shown its practical value. There is one objection, exercise as a curative has to be adapted to the condition of health, and hence in some cases it can not be utilized. It is as a prophylactic that it is of most value. This idea of exercise had been used by quacks for a long time in curing chronic cases.

Dr. BARTLETT reported under prevailing diseases some measles and some neuralgia of a peculiar character resembling pleuritis.

Dr. HOPKINS said that he thought that some explanation should

*Owing to lack of space Dr. Hopkins' paper is held over until next month.

be made in regard to the statements of Drs. Rochester and Cronyn that the formation of a membrane could occur without the precedence of inflammatory action, that statements such as had been made by these gentlemen should not be allowed to pass unchallenged, that acquiescence in such opinions would jeopardize the reputation of the Association.

Dr. CRONYN said that the opinion which he was prepared to sustain was that which he advanced several years since in the Association that membranous croup and diphtheria were identical.

Dr. GOULD asked how the mucous membrane could be destroyed by other means than by inflammation.

Dr. CRONYN explained that the mucous membrane could be destroyed without inflammatory action, as an instance in point, typhoid fever in which the mucous membrane of the intestine is destroyed by a slow process of ulceration.

Dr. FOLWELL said that in looking over Virchow and other authorities, he saw that the croupous deposit was formed on the epithelium of the mucous membrane.

Dr. CRONYN said it was a positive fact well understood by pathologists that it was impossible upon a mucous membrane to produce a fibrinous deposit.

Dr. HOPKINS said that it occurred to him that the exudation of croup was in some way the altered plasma of the blood which had become deposited in the vicinity, which had by some physiological, pathologial or histological process exuded through the mucous membrane and there become a false membrane.

Dr. CRONYN said that it would be found that when such membrane was formed that the mucous membrane would be absent. He read an extract from the paper of Dr. Semple upon the subject.

Dr. BARTLETT said that it seemed to him of the greatest possible importance that there should be no mistake between these two diseases. He concluded that there seemed to be a marked difference between diphtheria and croup. In a paper which he read upon this subject in this Association, he advised the employment of a blister upon the back of the neck. He thought it of great importance that the distinction should be recognized in order that we may know what treatment to employ.

He asked the opinion of the members present as to the reliability of the vaccine quills furnished by New York and other vaccine stations, and whether the crusts were not better.

Dr. O'Brien said that Dr. Foster of New York, would not send out bovine crusts except by urgent request, as he considered them unreliable; that all experienced in the use of bovine virus know this to be the case. He had always used bovine lymph, on the quill, furnished by Dr. Frank P. Foster of New York, and had always found it reliable. When the virus, on the quill, is of sufficiently recent issue, success should follow its use, if it has not been impaired by exposure to heat or moisture. It frequently happens that from inattention to some small, but important point, failure follows its use, to illustrate this he remarked that he had on several occasions successfully vaccinated patients when other physicians had failed to do so, from a part of the same quill used by them.

Quite an animated discussion followed as to the relative merits of quills and crusts humanized and non-humanized virus, during which the Secretary said he could endorse the statement of Dr. O'Brien as to the successful use of quills said to be inert.

On motion, the Society adjourned.

